# Morphometric brain changes during aging: Results from a Brazilian necropsy sample

**DOI:** 10.1590/S1980-57642010DN40400013

**Published:** 2010

**Authors:** Renata Eloah de Lucena Ferretti, Wilson Jacob-Filho, Lea Tenenholz Grinberg, Renata Elaine Paraízo Leite, José Marcelo Farfel, Claudia Kimie Suemoto, Paulo Hilário Nascimento Saldiva, Sérgio Rosemberg, Carlos Augusto Pasqualucci, Ricardo Nitrini

**Affiliations:** 1 Brazilian Brain Bank of the Aging Brain Study Group - Laboratory of Medical Investigations 22 (LIM 22).; 2 Division of Geriatrics, University of São Paulo Medical School, São Paulo SP, Brazil.; 3 University of ABC.; 4 Department of Pathology, University of São Paulo Medical School, São Paulo SP, Brazil.; 5 Department of Neurology, University of California, San Francisco.; 6 São Paulo Autopsy Service;; 7 Department of Neurology, University of São Paulo Medical School, São Paulo SP, Brazil.

**Keywords:** aging, brain/anatomy, cephalometry

## Abstract

The present study aimed to establish the morphometric brain changes during aging in a necropsy series from Brazil and determine whether sexual dimorphisms interfere in these changes.

**Methods:**

A cross-sectional study was conducted at the São Paulo Autopsy Service in Brazil where, after informed consent, data was gathered from next of kin interview with reference to clinical status prior to death. Brain weight, volume and density measurements were taken and then adjusted for head circumference. Descriptive statistics and tests of hypothesis and correlations were applied, considering a p-value of 0.05.

**Results:**

414 subjects, mostly men (60.4%), with a mean age of 67.1 years, were included. The mean brain weight of the sample was 1219.2g±140.9and mean volume was 1217mL±152.3. The mean brain density of the sample was 1.0g/mL±0.09. Values differed between males and females in terms of weight and volume. Brain weight decreased during aging by about 45g per decade (r= –0.300; p<0.01) and volume by about 43mL (r= –0.278; p<0.00). Mean density of the sample was 1.0 g/mL in both genders.

**Conclusions:**

Brain weight and volume (with or without corrections) decreased during aging, and these reductions were more pronounced in women. Density remained unchanged for both genders. Further studies are needed to investigate factors associated to these reductions.

## Abstract (pt)

O presente estudo buscou identificar quais as alterações morfométricas encefálicas durante o envelhecimento, em uma série de casos necropsiados do Brasil, segundo gênero.

**Métodos:**

Um estudo, em corte transversal, foi realizado no Serviço de Verificação de Óbitos de São Paulo onde, após consentimento informado, os dados foram coletados por meio de uma entrevista clínica com familiares em relação ao período anterior à morte. A massa e o volume encefálicos, assim como a densidade encefálica foram aferidas e ajustadas para o perímetro cefálico. Os dados foram analisados por meio de estatística descritiva e testes de hipóteses foram aplicados, considerando um p-valor de 0,05.

**Resultados:**

Amostra composta por 414 indivíduos, em sua maioria do gênero masculino (60,4%) com idade média de 67,1 anos. A massa media encefálica da amostra foi de 1219,2g±140,9 e o volume médio da amostra foi de 1217 mL±152,3. A densidade media foi de 1,0 g/mL±0,09. Os resultados diferiram entre homens e mulheres, em relação a massa e ao volume. A massa encefálica diminui durante o envelhecimento em aproximadamente 45g por década (r= –0,300; p<0,00) e o volume reduz aproximadamente 43 mL (r= –0,278; p<0,00). A densidade media foi de 1,0 g/mL, em ambos os gêneros.

**Conclusões:**

Massa e volume encefálicos (com e sem correção) diminuem durante o envelhecimento, sendo a redução mais pronunciada nas mulheres. A densidade global mantém-se estável em ambos os gêneros. Outros estudos são necessários para investigar os fatores associados às reduções.

Aging does not seem to be a uniform process as it is modulated by various intrinsic and extrinsic factors. It affects the entire human body but the Central Nervous System is subject to greatest impact due to its difficulty in recovering from injuries.

Neurodegenerative diseases are increasingly common during aging with a trend toward rising incidence and prevalence figures up until 2050 as populations age.^1^ These conditions lead to a great impact on public heath as these diseases cause impairments and poor quality of life. Most studies are currently aimed at gaining a better understanding of the physiopathology of aging but there is an evident lack of studies concerning the changes in brain structure and functioning during aging and related to senescence alone.

Some studies have described changes in the human brain. Greater understanding of human brain senescence would be valuable along with dissociating changes associated to neuropathological conditions from those decurrent from normal ageing.

Investigations have used two different methodological approaches: neuroimaging and necropsy studies. It has been cited that neuroimage technology currently seems to be the most common, and often the only, methodological approach available. However, better visualization of the brain can only be possible through necropsy studies, as these confer the real state of the structure. Postmortem studies are highly reliable, provided limitation factors can be controlled, as they allow better structure identification.^1,2^

Independently of method, studies based purely on senescence without disease are essential, especially in large population-based samples. According to DeCarli et al.,^3^ most studies have been based on small samples of individuals and have used very rigorous criteria for the inclusion of individuals, limiting the understanding of aging to its association with morbidity.^3^

It would be of a high interest to study a large population sample of elderly subjects using direct observation by necropsy methodology. Thus, the aim of the present study was to investigate the morphometric brain changes during aging through a necropsy study in Brazil.

## Methods

The present cross-sectional study is part of the Aging Brain Project of the Brain Bank of the Brazilian Aging Brain Study Group (BBBABSG).^4^ The study was approved by the Research Ethics Committee of the University of São Paulo and the National Ethics Committee, and complies with the Federal requirements for research involving human subjects.^5-7^

Data was collected at the mortuary service called the São Paulo Autopsy Service (SPAS), a public organ responsible for all necropsies in the metropolitan area of São Paulo. In Brazil, necropsies are compulsory for all individuals who die without a defined cause of natural death.

This study follows the methodological approaches of the Brazilian Brain Bank and only related procedures will be described here. The complete BBBABSG procedures have been described elsewhere.^4,8^

### Sample characterization and inclusion procedures

The sample comprised deceased subjects aged 50 years and older at time of death sourced from the SPAS. Corpses arrive at the SPAS 24 hours a day for autopsy procedures and a legally authorized subject, most often a relative (so-called next – of – kin “NOK”), must come to the service for the funeral proceedings and to authorize release of the body.

Upon NOK’s arrival, a team of gerontologists was responsible for receiving the NOK and collecting Informed Consent Forms. After the initial contact, families were taken to a private room where a clinical interview was performed. Sometimes NOK were not available and in these cases an informant, known as Collateral Source (CS), able to give accurate information about the deceased was involved instead. Information gathered from CSs has proven reliable (regarding cognitive status of the subject) whenever informants have had previous close contact with the subject.^9-15^

Cases that fulfilled the inclusion criteria were included in the sample. Inclusion criteria consisted of:

[a] subjects aged 50 years and older who died of natural causes and were at the SPAS;
[b] those who had an NOK or a competent CS to give reliable information and
[c] individuals without cognitive decline. The remaining inclusion and exclusion criteria of the BBBABSG were also respected.^4,8^

**Figure 1:**
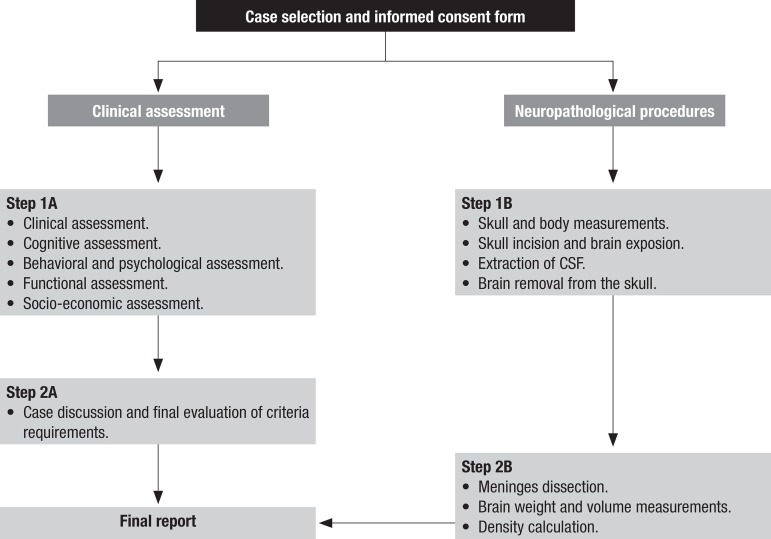
Flowchart of the BBBABSG. São Paulo, 2010.

The flowchart of procedures is shown in Figure 1 and allows better understanding of the complete process.

### Clinical assessment

All the clinical interviews were done while the NOK was waiting for the release of the body. Clinical interviews consisted of a battery of instruments preceded by an anamnesis. The aim of the interview was to:

[i] investigate and determine the clinical, cognitive, behavioral and functional status of the subject during their lifetime;
[ii] determine whether there were clinical conditions associated to exclusion criteria;
[iii] have NOK sign the informed consent form.

As the sample comprised subjects with no cognitive decline, all cases were CDR zero, i.e. “without dementia”.

Interviews were done by a team of trained nurses supervised by a geriatric nurse/ gerontologist. Clinical Nurse Specialists are able to identify and stage dementia in a very reliable manner through the use of the CDR.^16^

After the interview, the case was discussed in a consensus format, which encompassed an interdisciplinary full analysis of the case by a neurologist, a geriatrician and the gerontologists, aiming to reach the Best Estimated Diagnosis.

### Neuropathological procedures

Brains were collected during the autopsy procedure. All the autopsies were performed by the SPAS pathologist.

**Figure 2:**
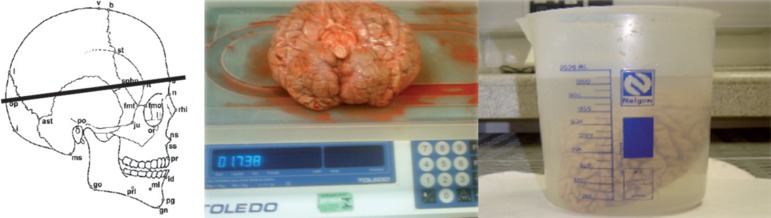
Head circumference, weight and volume measurements. (Photos from the BBBABSG’s collection).

Before the necropsy began, the head circumference measurement was taken with the skull still closed. Head circumference is important for brain measurement adjustments and interpretation, since it is related to a secular effect. This measurement was obtained by the use of inelastic metric tape that was placed around the head of the subject, so as to measure the distance between the cephalic markers “*glabela*” and “*opstocraniun*”, as illustrated in Figure 2.

During autopsy, before the removal of the brain, the cerebral spinal fluid was extracted. Secondly, brain weight and volume were measured. Brain weight (g) was measured by using a precision scale and the volume measurement (mL) was obtained by the Archimede’s principle. The whole procedure took about thirty minutes.

Density value was obtained, dividing the weight measured by the volume obtained.

### Statistical analysis

The sample was stratified into two groups according to gender and then divided into subgroups according to age. Age groups were split into 10-year class intervals. All data was adjusted by the variable “head circumference” in order to avoid secular effect.

Central tendency tests were used for descriptive statistics. The hypothesis tests included the t test (t) and Chi-squared (χ^2^) test to verify statistical significance in continuous and nominal data, respectively. Correlations were done by Pearson’s (r) and Spearman’s (r) correlation tests, for continuous data and ordinal data, respectively.

## Results

All cases which had brain measurements (head circumference, weight and volume) and clinical interview data available were included in the sample, which spanned the period of 2005 and 2006. Corpses arrived at the SPAS with a mean Postmortem Interval (PMI) of 14.8 hours (±4.6h).

The sample comprised 414 cases with subjects aged 50 years and older (50 to 98 years) with a mean age of 67.1 years (±10.9 years), and were mostly males (60.4%). Mean age was higher among women (68.5±11.9) than among men (66.2±10.2) with a p-value of 0.001. With regard to literacy, the mean years of education of the sample was 4.6±3.6 years, with higher schooling among men (5.0±3.6) than women (3.8±2.2), and a p-value of 0.001. Subjects were mostly Caucasian (69%) and 70.5% of the cases had low socio-economic level.

The majority of the NOK reported that their relative had medical conditions during life (86.5%), but none of the subjects reported history of cognitive decline, as this was the main exclusion criteria of the study.

The mean brain weight of the sample was 1219.2g± 140.9 and the mean volume was 1217mL±152.3. The mean brain density of the sample was 1.0g/mL±0.09. Values differed between males and females for weight and volume.

Brain weight decreases during aging at approximately 45g per decade (r= –0.300; p<0.01). Considering mean weight, men showed higher values than did women: 1264.4 ±127.2g and 1149.6±135.4g, respectively. After adjusting for head circumference, it was observed that brain weight decreased during aging in both genders (r = –0.273; p<0.01). The mean corrected weight was 21.7 g/cm (±2.3), 20.9 g/cm (±2.4) among women and 22.2 g/cm (±2.1) among men.

Volume also decreased with aging. Brain volume decreased during aging by about 43mL per decade (r= –0.278; p<0.01). Considering mean volume, men showed higher values than did women: 1260.0±121.4g and 1145.6 ±128.3mL, respectively. After adjusting for head circumference, it was observed that brain volume decreased during aging in both genders (r= –0.264; p< 0.01). The mean corrected volume was 21.6 mL/cm (±2.5), 20.8 mL/cm (±2.3) among women and 22.1 mL/cm (±2.6) among men.

Global brain density value proved stable during aging in both genders. Mean density of the sample was 1.0 g/mL.

Part of the sample was composed of subjects with no medical condition diagnosed during lifetime, i.e. NOK reported they had no disease. This subgroup contained 65 individuals (15.7%). Comparison of males and females belonging to this group revealed some differences compared to the whole sample. Men (52 cases) had a mean age of 63.4 years±10.7(ranging from 50 to 80 years) and showed a mean brain weight of 1278.6g, a mean brain volume of 1279.7 mL. Women (13 cases) had a mean age of 65.9 years±13.4(ranging from 50 to 84 years) and showed a mean brain weight of 1177.3g, and a mean brain volume of 1165.4 mL.

## Discussion

Results from the present study are consistent with those reported in the literature to date, concerning morphometric brain changes during aging. Previous studies have demonstrated that brain shrinks with age, resulting in visible macroscopic changes in cephalic tissue,^1-3,17-22^ but this only represents the end stage of a complex phenomenon.^22^

Some studies have described that mean brain weight is 9% to 12% higher in men than in women.^17,21,23^ This was also observed in the present series where the difference was around 11.1%. The Brazilian mean brain weight was found to be very similar to the values obtained from a Venezuelan study, but lower than values obtained in North-American and European samples. Notably however, the Venezuelan sample was not adjusted for head circumference.^20,21,24^

Another well-documented difference between genders is that brain weight and volume diminish as age increases, and this decrease is higher among man than in women,^1,21,25^ may be due to sample characteristics. In the present sample, it was observed that the decrease in weight and volume was more pronounced in women. This could possibly be explained by increased cognitive decline with aging. Since cognitively-impaired subjects were excluded from the sample, the prevalence of cognitively normal men was expected to be higher compared to women who tend to be older and more cognitively impaired. Furthermore, for decades commonly associated to lower weight and volume values, the number of women was higher than men because the latter tended to die earlier.

The influence of gender on cerebral changes is well known. The fact that brain weight and volume values during aging were more pronounced in women can be explained by hormonal changes inherent to gender. It has been documented that estrogen confers some protection. However, after menopause a drop in the hormone makes brain weight and volume reductions even more marked in women, thus making this equal in female and male genders. Also, there is a greater impact due to sexual dimorphism from the age of 60 and older.^3^

Real time measurements of brain weight and volume can be obtained by direct observation, which makes necropsy “a gold standard” in brain studies compared to neuroimaging.^26^ Archimede’s principle for volume measurement is simple and much more precise for this analysis than that obtained by neuroimaging methods.^21^

On the other hand, there are some limitations in postmortem studies in that the analysis might be influenced by various factors, such as PMI, the timing for measurements, tissue fixation methods and some medical conditions that could cause brain edema.^2,17,26,27^ In our sample, PMI was lower compared to PMI of other series.^4,28^

Special attention has been dedicated to inclusion criteria. Subjects whose cause of death could be related to clinical conditions, deemed capable of interfering in the tissue analysis, were excluded. Moreover, weight and volume measurements were taken as soon as possible after skull opening and brain removal. This has eliminated artifacts related to tissue fixation.

Brain weight and volume vary according to height, as explained by the secular effect theory.^29,30^ The secular effect holds that the increase in human height over the last century was accompanied by an increase in brain weight and volume,^17,25,27^ thus justifying corrections in measurements to head circumference. Also, corrections are also extremely important in order to avoid variability in height: skull size ratios among subjects.^31^ In this series, head circumference was used instead of height, for the adjustment procedures. Head circumference has been considered a very reliable measure in studies that have carried out correction of brain weight and volume.^31,32^ The use of head circumference eliminates the impact of senility on height reduction, given some pathological conditions may reduce height besides senescence such as immobility, contractions etc. that could consequently interfere in the accuracy of the correction. Brain weight and volume were corrected for head circumference. Despite correction, both weight and volume (total values and corrected) decreased with age in both genders.

Given global density is the product of a direct relationship between brain weight and volume and, considering that weight and volume both diminish with age, density appears to be stable. Previous studies investigating density have used neuroimaging methods and described regional changes in the density of white and gray matter, but not global changes.

Last but not least, the sample comprised subjects without cognitive decline, demonstrating that during senescence there are some changes in brain morphometry but these are not sufficient to determine cognitive impairment *per se*, probably due to brain cognitive reserve which is related not only to brain size but principally to its neuronal activity.^33,34^

Concluding, the present study describes morphometric brain changes in a large necropsy series from Brazil and highlights that brain weight and volume (with or without corrections) decreased during aging, in contrast to unchanged density, and these reductions were more pronounced in women. Other studies are needed to investigate factors associated to these reductions.

## Figures and Tables

**Figure 3:**
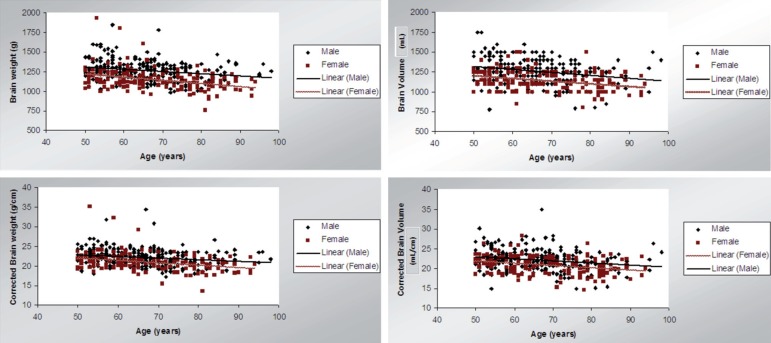
Scatterplot of brain weight and volume (total and corrected values) during aging, by gender.

**Figure 4:**
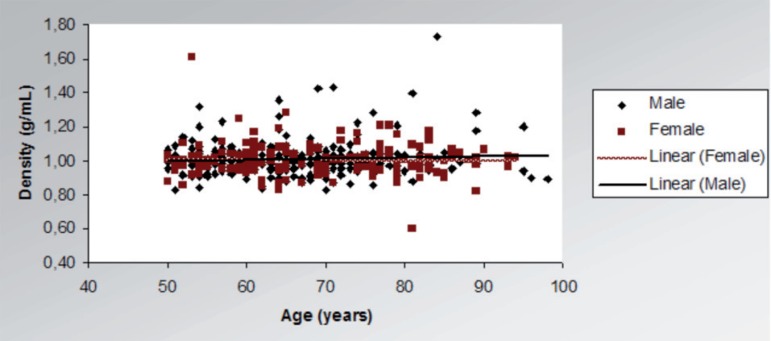
Distribution of brain density during aging, according to gender.
